# Endotoxin-induced cytokine and chemokine expression in the HIV-1 transgenic rat

**DOI:** 10.1186/1742-2094-9-3

**Published:** 2012-01-04

**Authors:** Natasha F Homji, Xin Mao, Erik F Langsdorf, Sulie L Chang

**Affiliations:** 1 Institute of NeuroImmune Pharmacology, Seton Hall University, South Orange, NJ, 07079, USA; 2 Department of Biological Science, Seton Hall University, South Orange, NJ, 07079, USA

**Keywords:** HIV-1 transgenic rat, endotoxin tolerance, cytokines, chemokines

## Abstract

**Background:**

Repeated exposure to a low dose of a bacterial endotoxin such as lipopolysaccharide (LPS) causes immune cells to become refractory to a subsequent endotoxin challenge, a phenomenon known as endotoxin tolerance (ET). During ET, there is an imbalance in pro- and anti-inflammatory cytokine and chemokine production, leading to a dysregulated immune response. HIV-1 viral proteins are known to have an adverse effect on the immune system. However, the effects of HIV-1 viral proteins during ET have not been investigated.

**Methods:**

In this study, HIV-1 transgenic (HIV-1Tg) rats and control F344 rats (n = 12 ea) were randomly treated with 2 non-pyrogenic doses of LPS (LL) to induce ET, or saline (SS), followed by a high challenge dose of LPS (LL+L, SS+L) or saline (LL+S, SS+S). The gene expression of 84 cytokines, chemokines, and their receptors in the brain and spleen was examined by relative quantitative PCR using a PCR array, and protein levels in the brain, spleen, and serum of 7 of these 84 genes was determined using an electrochemiluminescent assay.

**Results:**

In the spleen, there was an increase in key pro-inflammatory (IL1α, IL-1β, IFN-γ) and anti-inflammatory (IL-10) cytokines, and inflammatory chemokines (Ccl2, Ccl7, and Ccl9,) in response to LPS in the SS+L and LL+L (ET) groups of both the HIV-1Tg and F344 rats, but was greater in the HIV-1Tg rats than in the F344. In the ET HIV-1Tg and F344 (LL+L) rats in the spleen, the LPS-induced increase in pro-inflammatory cytokines was diminished and that of the anti-inflammatory cytokine was enhanced compared to the SS+L group rats. In the brain, IL-1β, as well as the Ccl2, Ccl3, and Ccl7 chemokines were increased to a greater extent in the HIV-1Tg rats compared to the F344; whereas Cxcl1, Cxcl10, and Cxcl11 were increased to a greater extent in the F344 rats compared to the HIV-1Tg rats in the LL+L and SS+L groups.

**Conclusion:**

Our data indicate that the continuous presence of HIV-1 viral proteins can have tissue-dependent effects on endotoxin-induced cytokine and chemokine expression in the ET state.

## Background

The bacterial endotoxin, lipopolysaccharide (LPS), is a well-characterized glycolipid component of the cell wall of gram-negative bacteria [1-3]. LPS is a model molecule commonly used to study the inflammatory responses caused by exposure to bacteria, in particular, the induction and actions of inflammatory cytokines and chemokines [4-6]. An inflammatory response involves a balance between the production of pro-inflammatory cytokines and chemokines and the subsequent production of anti-inflammatory cytokines [7]. An imbalance in this mechanism can lead to disastrous immune system-related consequences. Tight control of pro-inflammatory cytokine production is necessary in order to protect against septic shock. An imbalance in this regulatory mechanism can also lead to the development of endotoxin tolerance (ET) [8-14]. In ET, repeated exposure to minute amounts of an endotoxin, like LPS, causes immune cells, such as macrophages and monocytes, to become refractory to a subsequent high-dose endotoxin challenge [7,11,13,15-17]. On re-exposure to an endotoxin, when the animal is in an ET state, there is an increase in production of anti-inflammatory cytokines and a decrease in production of pro-inflammatory cytokines in comparison to a single exposure to the endotoxin [14]. ET is known to resemble immunosuppression in many aspects reported in patients with sepsis or non-infectious systemic inflammatory response syndrome (SIRS) [18]. While ET initially protects against severe infection and tissue damage by overt inflammatory response, however the immune dysregulation observed in ET and in SIRS patients is associated with greater propensity to succumb to nosocomial infections [18,19].

The Human immunodeficiency virus-1 (HIV-1) is characterized by very rapid viral replication. The virus is subsequently transported to the lymphoid organs and the central nervous system (CNS). A very strong cellular and humoral immune response is evoked in the host within a few weeks [20], after which there is a clinical latency period, sometimes for years, followed by rapid clinical deterioration [21]. It is believed that the continued presence of HIV-1 viral proteins plays a role in the clinical progression of HIV-1 infection to full-blown AIDS [22-26].

Since 1996, highly active anti-retroviral therapy (HAART) has resulted in a dramatic improvement in the health and longevity of HIV-infected individuals [27]. However, HAART drugs are limited in their capacity to enter the CNS and other organs that are protected by tight endothelial barriers. Thus, in this post-HAART era, the clinical challenge is to identify the biological and physiological changes that occur due to the persistent presence of HIV-1 viral proteins in the host even when active viral replication is arrested [28,29].

Some HIV-1 viral proteins have been shown to affect the inflammatory response by altering the production of cytokines. For example, the HIV-1 Tat protein can alter the LPS-induced production of IFN-β and IL-6 in blood monocytes/macrophages [30], and HIV-1 Vpr suppresses IL-12 production in human monocytes [31]. However, the effects of HIV-1 viral proteins on immune function during a state of ET has not been examined.

The HIV-1 transgenic (HIV-1Tg) rat model was developed with a functional deletion of the *gag *and *pol *genes in the HIV-1 genome. It is, however, under the control of the viral promoter and expresses seven of the nine HIV genes [32]. Thus, in the HIV-1Tg rat, there is no HIV-1 replication, but other HIV-1 viral proteins are expressed [32]. We have shown that, like HIV-1 infected patients, the HIV-1Tg rat is immunodeficient. LPS-induced leukocyte-endothelial adhesion (LEA) is greatly attenuated in the HIV-1Tg rat [33]. In addition, the HIV-1Tg rat shows signs of wasting and dies at a younger age even though there is no growth retardation and no sign of anorexia throughout its life span [34]. These rats also have decreased alveolar macrophage zinc levels and phagocytosis [35]. The HIV-1Tg rat model, thus, exhibits some of the characteristics of HIV-1 infected patients given HAART [36-39].

There is an association between chronic HIV infection and elevated plasma endotoxin levels. Innate immune responses, which are dysregulated in ET, are also altered in HIV infection [40]. The cytokines/chemokines modulated during ET play a role in HIV infection, replication (not applicable for our model) and pathogenesis. Toll-like receptor 4 (TLR4) mediates gram-negative bacteria activated signaling and significant changes in this receptor's level is directly correlated with HIV infection [41]. TNF-α upregulation leads to HIV-induced cytotoxicity [42]. Enhanced levels of Ccl2 in HIV-1 patients have been associated with HIV-1-associated dementia [43]. The Center for Disease Control (CDC) has identified HIV-1 infection as a major reason for the increase in incidence of sepsis [44]. Opportunistic infections are a common feature in HIV-1 positive patients, who have a compromised immune status [45]. ET leads to a similar immunosuppressed state. Identifying the mechanism by which ET affects an already immune-compromised system, as in HIV-1 infection, could provide valuable information of clinical relevance. We hypothesized that the continuous presence of HIV-1 viral proteins alters the systemic immune response to bacterial endotoxins in terms of pro- and anti-inflammatory cytokine and chemokine expression, and that this altered immune response is exacerbated when the animal is in an ET state. Specifically, we hypothesized that the production of pro-inflammatory cytokines is diminished and anti-inflammatory cytokine production is enhanced in the HIV-1Tg rat rendered endotoxin tolerant. To test this hypothesis, in this study, we examined the expression of an array of cytokines, chemokines, and their receptors in the serum, spleen, and brain of an endotoxin tolerant HIV-1Tg rat model in response to an LPS challenge.

## Methods

### Animals

Adolescent male Sprague-Dawley HIV-1 transgenic (HIV -1Tg) rats and age-matched Fisher/NHsd 344 (F344) control rats were purchased from Harlan Laboratories (Indianapolis, IN), and were delivered on post-natal day 28. The animals were group-housed immediately upon arrival, and stayed in group cages during the experiment. The animals were maintained in an environment of controlled temperature (21-22°C) on a 12-h light/12-h dark illumination cycle, with lights-on set at 7:00 AM. Food and tap water were provided *ad libitum*. The experimental protocol was approved by the Institutional Animal Care and Use Committee (IACUC) at Seton Hall University, South Orange, NJ.

### Lipopolysaccharide (LPS) administration

Dosing solutions of LPS were prepared in saline. In our preliminary studies using Harlan Sprague Dawley rats, we found that two intraperitoneal (i.p) injections with a non-pyrogenic dose (250 μg/kg ea) of LPS, administered 9-12 hrs apart, was the lowest dosage regimen that would cause endotoxin tolerance and inhibit the production of IL-1β, TNF-α, and IL-6 in response to a subsequent challenge with a significantly higher dosage of LPS [1, 4, 8, 16, or 32 mg/kg] (data not shown). In this study, HIV-1Tg and F344 rats (n = 12 ea, 19-20 wks old) were randomly assigned to four experimental groups (n = 3 animals/group). At 8:00 AM and 5:00 PM on Day 1, Groups 1 and 2 received two i.p. injections of 250 μg/kg LPS each (LL); Groups 3 and 4 received two i.p. injections of saline (SS). At 8:00 AM on Day 2, Group 1 received one i.p. injection with 5 mg/kg LPS (LL+L); Group 2 received one i.p. injection with saline (LL+S); Group 3 received one i.p. injection with 5 mg/kg LPS (SS+L); and Group 4 received one i.p. injection with saline (SS+S). The dosage of 5 mg/kg for the subsequent LPS injection was chosen based on previous studies using the HIV-1Tg rat model [46]. Two hours following the last injection, the brains, spleens, and blood were collected for RNA, protein, and serum preparation.

### Protein extraction from the brain and spleen

Protein extracts were prepared from approximately 100 mg of brain and spleen tissue in a Tris lysis buffer containing 20 mM Tris, pH 7.5, 1 mM EDTA, 1 mM EGTA, and 1 M NaF (all from Sigma Aldrich, St. Louis, MO), and 1 tablet of Protease Inhibitor (Roche, Mannheim, Germany). The tissues were disrupted using the Branson Sonifier 250 (VWR, Radnor, PA) with 15 sec bursts, a duty cycle setting of 40% (0.4 sec burst/0.6 sec pause), and an output of 4. The concentration of protein from each of the tissue types was determined using the ProStain assay kit (Active Motif, Carlsbad, CA), and measured as fluorescence intensity against a BSA standard curve with the Spectra Max Gemini (Molecular Devices, Sunnyvale, CA).

### Measurement of inflammatory cytokines

Protein levels of IL-1β, KC/GRO, IL-4, IL-5, TNF-α, IFN-γ, and IL-13 were determined in undiluted serum and in extracts from 200 μg of brain and spleen using a 96-well inflammatory cytokine kit [MesoScale Discovery (MSD), Gaithersberg, MD]. Measurement of electrochemiluminescent signal intensity was determined on the SECTOR 2400 instrument (MesoScale Discovery, Gaithersberg, MD). Calibrator solutions were diluted 4-fold over a concentration range of 40,000 pg/mL to 9.8 pg/mL.

### RNA isolation and preparation of cDNA

Total RNA was extracted from brain and spleen homogenates using TRIZOL (Invitrogen, Carlsbad, CA). The extracts were then treated with Ambion^® ^TURBO DNA-*free*™ (Ambion, Austin, TX) to remove contaminating DNA, and harvested using a RNeasy mini kit (Qiagen, Valencia, CA). RNA quality and quantity were assessed using a Nanodrop spectrophotometer. Equal amounts of RNA from each sample were then converted into first-strand cDNA using a RT2 First Strand Kit (SA Biosciences, Frederick, MD),

### Real-time PCR array

Detection and quantification of gene expression were performed using a Rat Inflammatory Cytokines and Receptors PCR Array and RT^2 ^SYBR Green Fluorescin qPCR Master (SA Biosciences, Frederick, MD) according to the manufacturer's instructions. This kit was chosen because it includes diverse genes important in immune responses, including genes encoding CC chemokines (n = 16), CXC chemokines (n = 9), interleukin cytokines (n = 14), other cytokines (n = 11), chemokine receptors (n = 15), and cytokine receptors (n = 11), as well as other genes involved in the inflammatory response (n = 8).

Real-time PCR was performed using an ABI Prism 7900HT Fast Detection System (Applied Biosystems, Foster, CA). Each 10 μL reaction was performed in a 384-well format PCR array. The PCR mix was denatured at 95°C for 10 min before the first PCR cycle. The thermocycler parameters were 95°C for 10 min, followed by 40 cycles at 95°C for 15 s, and 60°C for 1 min.

### PCR array data analysis

In order to be able to compare different PCR array results, the threshold and baseline values were set manually, according to the manufacturer's instructions, and the resulting threshold cycle value (CT) data were uploaded into the data analysis template on the manufacturer's website http://www.sabiosciences.com/pcr/arrayanalysis.php. RNA expression of each gene was normalized using five housekeeping genes as controls. The relative expression of each gene, compared with the expression in the control group, was calculated on the website using the ΔΔCT method. A difference was considered significant at *p *< 0.05. In the expression studies, a gene was considered differentially regulated if the difference was > 2-fold compared with the control, and markedly differentially regulated if the difference was > 10-fold [47-52]. Each reported value represents the mean increase or decrease of mRNA expression relative to the levels for the controls from three biological replicates.

### Statistical analysis

Inflammatory cytokine protein level data in this study are presented as the mean ± SD. Statistical analysis was done using Graphpad Prism 5.0. Differences among treatment groups were analyzed by a one-way ANOVA, followed by a Newman-Keuls *post hoc *test. The difference in the basal levels of cytokines/chemokines in the brain, spleen, and serum between F344 and HIV-1Tg rats was determined using the *Student's t*-test.

## Results

### Cytokine and chemokine protein expression in the serum, brain, and spleen of HIV-1Tg rats during endotoxin tolerance (ET)

The protein levels of IL-1β, KC/GRO, IL-4, IL-5, TNF-α, IFN-γ, and IL-13 were determined in the brain, spleen, and sera of HIV-1Tg and F344 rats rendered ET (LL), then challenged with either LPS (LL+L) or saline (LL+S), using an electrochemiluminescent (MSD) assay (Table 1, 2, 3). Saline-treated animals were used as controls (SS+S). There was no difference in the basal levels (SS+S group levels) of the cytokines/chemokines measured in the serum in the two strains of animals (Table 1). In the F344 serum, the levels of the pro-inflammatory cytokine, IFN-γ, and the anti-inflammatory cytokines, IL-4 and IL-13, in the single exposure group (SS+L) were significantly greater than in the control group (SS+S), and significantly less in the ET group (LL+L) than in the single exposure group (SS+L). In the HIV-1Tg serum, the levels of the pro-inflammatory cytokines, IL-1β, TNF-α, and IFN-γ, and the anti-inflammatory cytokines, IL-4 and IL-13, in the single exposure group (SS+L) were significantly higher than in the control group (SS+S), and significantly lower in the ET group (LL+L) than in the single exposure group (SS+L) [Table 1].

**Table 1:** Cytokine/Chemokine Profiles in F344 and HIV-1Tg Rat Serum

Cytokines and Chemokines in the Serum with LPS Treatment							
	**Protein (pg/ml, ± SD)**						

**F344**	IL-1β	KC/GRO	IL-4	IL-5	TNF-α	IFN-γ	IL-13
	
	Interleukin 1 beta		Interleukin 4	Interleukin 5	Tumor necrosis factor - alpha	Interferon gamma	Interleukin 13

SS+S	83.0(± 21.3)	1294.8(± 589.7)	9.5(± 0.6)	116.0(± 23.7)	42.7(± 6.1)	35.2(± 7.2)	27.5(± 1.1)

SS+L	272.1(± 145.1)	7153.2(± 2779.8)**	15.5(± 1.7)***	348.7(± 202.4)	7531.8(± 7897.0)	138.6(± 46.2)**	42.3(± 9.5)**

LL+S	80.4(± 42.4)	780.9(± 846.6)	10.3(± 51.2)	103.6(± 32.6)	51.2(± 12.4)	56.5(± 19.6)	22.5(± 1.1)

LL+L	125.1(± 23.5)	3293.5(± 680.6)*	**11.1(± 1.1)^^**	210.0(± 56.7)	88.2(± 25.5)	**61.8(± 18.9)^^**	**26.5(± 2.1)^**

**HIV - 1Tg**	IL-1β	KC/GRO	IL-4	IL-5	TNF-α	IFN-γ	IL-13
	
	Interleukin 1 beta		Interleukin 4	Interleukin 5	Tumor necrosis factor - alpha	Interferon gamma	Interleukin 13

SS+S	85.2(± 31.6)	1907.4(± 978.9)	10.7(± 2.0)	154.7(± 83.6)	45.2(± 11.4)	67.8(± 32.2)	26.8(± 0.5)

SS+L	307.4(± 72.9)***	7002.2(± 361.0)***	14.4(± 2.5)	258.3(± 37.3)	2366.7(± 924.4)***	122.3(± 13.4)*	36.2(± 6.6)*

LL+S	47.9(± 7.4)	327.6(± 110.5)	8.0(± 1.4)	29.7(± 34.3)	42.8(± 7.0)	18.3(± 24.9)	22.3(± 1.1)

LL+L	**128.6(± 3.0)^^^**	**3051.3(± 1215.7)^^^**	**11.3(± 0.8)^^**	189.1(± 121.1)	**113.4(± 51.8)^^^**	**71.1(± 9.1)^**	**27.9(± 4.2)^**

* *P *< 0.05, ** *P *< 0.01, *** *P *< 0.001 greater than SS+S control; ^ *P *< 0.05, ^^ *P *< 0.01, ^^^ P < 0.001 less than SS+L; statistically significant changes are bolded

**Table 2:** Cytokine/Chemokine Profiles in the F344 and HIV-1Tg Rat Brain

Cytokines and Chemokines in the Brain with LPS Treatment							
	**Protein (pg/ml, ± SD)**						

**F344**	IL-1β	KC/GRO	IL-4	IL-5	TNF-α	IFN-γ	IL-13
	
	Interleukin 1 beta		Interleukin 4	Interleukin 5	Tumor necrosis factor - alpha	Interferon gamma	Interleukin 13

SS+S	42.2(± 8.8)	74.4(± 23.3)	ND	56.5(±)	ND	15.3(± 7.5)	4.4(± 1.8)

SS+L	178.4(± 162.1)	1325.3(± 1431.3)	13.5()	102.8(± 61.1)	30.9	20.5(± 11.9)	4.8(± 3.1)

LL+S	59.1(± 26.3)	112.6(± 35.6)	3.4	74.7	ND	14.7(± 7.0)	6.9(± 1.4)

LL+L	207.0(± 133.0)	1036.2(± 685.6)	2.9(± 1.3)	141.9(± 98.3)	ND	21.9(± 6.5)	5.3(± 2.3)

**HIV - 1Tg**	IL-1β	KC/GRO	IL-4	IL-5	TNF-α	IFN-γ	IL-13
	
	Interleukin 1 beta		Interleukin 4	Interleukin 5	Tumor necrosis factor - alpha	Interferon gamma	Interleukin 13

SS+S	37.3(± 17.1)	125.3(± 87.8)	ND	ND	ND	11.7(± 5.2)	5.1(± 0.4)

SS+L	361.7(± 104.6)*	2991.3(± 491.5)**	9.2(± 3.1)	164.2(± 35.3)	109.5	47.1(± 14.5)*	9.3(± 4.2)

LL+S	56.5(± 15.1)	128.1(± 61.6)	ND	ND	ND	8.9(± 5.9)	4.6(± 1.4)

LL+L	208.6(± 167.4)	**1312.9(± 1039.2)^**	8.1(± 1.6)	181.2(± 50.0)	27.3	**24.3(± 14.5)^**	7.3(± 3.7)

* *P *< 0.05, ** *P *< 0.01, *** *P *< 0.001 greater SS+S control; ^ *P *< 0.05, ^^ *P *< 0.01, ^^^ P < 0.001 less than SS+L; statistically significant changes are bolded

**Table 3:** Cytokine/Chemokine Profiles in the F344 and HIV-1Tg Rat Spleen

Cytokines and Chemokines in the Spleen with LPS Treatment							
	**Protein (pg/ml, ± SD)**						

**F344**	IL-1β	KC/GRO	IL-4	IL-5	TNF-α	IFN-γ	IL-13
	
	Interleukin 1 beta		Interleukin 4	Interleukin 5	Tumor necrosis factor - alpha	Interferon gamma	Interleukin 13

SS+S	1802.9(± 635.7)	264.7(± 122.7)	6.9(± 3.3)	119.5(± 20.0)	ND	34.5(± 13.6)	3.7(± 2.3)

SS+L	19030.7(± 11365.4)*	20065.6(± 11858.1)	50.7(± 16.0)**	344.4(± 83.9)**	2943.6(± 2752.3)	203.3(± 71.5)**	22.1(± 6.0)**

LL+S	3325.3(± 440.6)	506.6(± 205.0)	6.8(± 5.7)	120.0(± 28.8)	ND	32.5(± 28.1)	3.8(± 3.0)

LL+L	7898(± 1996.9)^	11818.7(± 9648.2)	**21.6(± 10.2)^^**	246.6(± 79.4)	228.7(± 19.6)	105.3(± 45.0)*^	14.2(± 6.1)

**HIV - 1Tg**	IL-1β	KC/GRO	IL-4	IL-5	TNF-α	IFN-γ	IL-13
	
	Interleukin 1 beta		Interleukin 4	Interleukin 5	Tumor necrosis factor - α	Interferon gamma	Interleukin 13

SS+S	1274.5(± 122.3)	361.1(± 187.7)	3.8(± 1.4)	107.7(± 33.0)	ND	10.8(± 4.8)	0.8(± 0.7)

SS+L	19951.8(± 4023.8)**	25812.5(± 3014.5)**	41.8(± 5.7)**	395.4(± 93.3)**	2755.4(± 1596.0)	296.9(± 83.4)**	32.0(± 4.9)*

LL+S	5087.1(± 1675.4)	961.8(± 89.1)	7.3(± 2.0)	178.4(± 57.2)	ND	19.8(± 2.6)	6.6(± 6.4)

LL+L	11443.1(± 7400)*^	**12754.4(± 11613.3)^**	27.3(± 12.6)*^	**239.4(± 77.1)^**	356.7(± 149.4)	151.5(± 77.6)*^	19.8(± 11.4)

* *P *< 0.05, ** *P *< 0.01, *** *P *< 0.001 greater than SS+S control; ^ *P *< 0.05, ^^ *P *< 0.01, ^^^ P < 0.001 less than SS+L; statistically significant changes are bolded

The basal levels of IL-4 and TNF-α in the F344 rat brain, and IL-4, IL-5, and TNF-α in the HIV-1Tg brain, were not detectable by electrochemiluminescent (MSD) assay. The basal levels of cytokines/chemokines that were detectable in the brain were similar in both strains of animals (Table 2). An LPS challenge dose (SS+L and LL+L) did not significantly alter any of the cytokine/chemokine levels in the F344 rat brain (Table 2). However, in the HIV-1Tg rat brain, the levels of the pro-inflammatory cytokines, IL-1β and IFN-γ, were significantly higher in the SS+L group versus the control. The level of the pro-inflammatory cytokine, IFN-γ, was significantly lower in the LL+L group compared to the SS+L group (Table 2).

The basal level of TNF-α was not detectable in the spleen of either the F344 or HIV-1Tg rats (Table 3). There was no significant difference in the basal levels of any of the cytokines/chemokines that were detectable in the spleen of the F344 and HIV-1Tg rats, with the exception of IFN-γ, which was significantly greater in the F344 rat spleen compared to that in the HIV-1Tg rat spleen (Table 3). In the F344 spleen, the levels of the pro-inflammatory cytokines, IL-1β and IFN-γ, and the anti-inflammatory cytokines, IL-4 and IL-13, in the single exposure group (SS+L) were significantly higher than in the control. In the F344 rats, the levels of the pro-inflammatory cytokines, IL-1β and IFN-γ, and the anti-inflammatory cytokine, IL-4, were significantly lower in the spleen of the LL+L group compared to the single exposure group (SS+L). In the HIV-1Tg spleen, the levels of the pro-inflammatory cytokines, IL-1β and IFN-γ, and the anti-inflammatory cytokine, IL-4, were significantly higher in the single exposure group (SS+L) and in the endotoxin tolerant group (LL+L) compared to the control. In the LL+L group of HIV-1Tg rats, the pro-inflammatory cytokines, IL-1β and IFN-γ, and the anti-inflammatory cytokine, IL-4, were significantly lower than in the single exposure (SS+L) group (Table 3).

### Expression profiles of cytokines, cytokine-receptors, and other inflammatory molecules in the brain and spleen of the HIV-1Tg rat following LPS treatment

**Figure 1:**
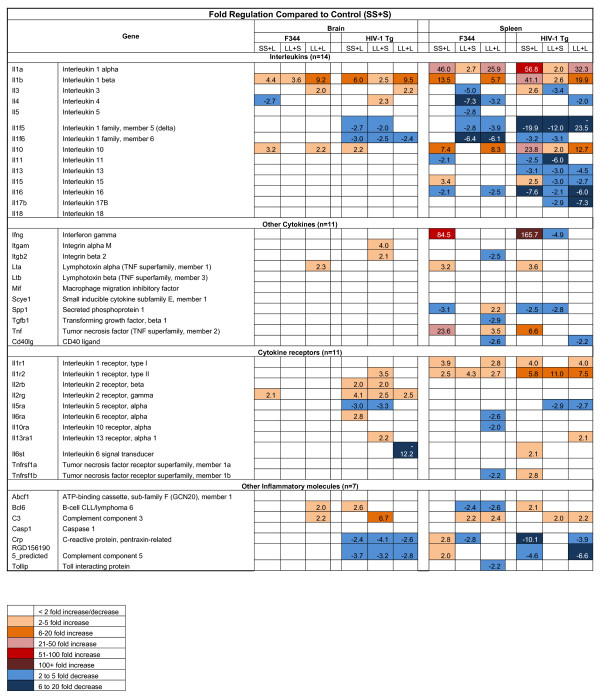
**Cytokine Expression in the Brain and Spleen of F344 and HIV-1Tg Rats**.

The gene expression profiles of an array of cytokines, cytokine receptors, and other inflammatory molecules were determined in the brain and spleen of the three LPS treatment groups (SS+L, LL+S, and LL+L) and compared to the control group (SS+S) of both the HIV-1Tg and F344 rats (Figure 1). In the F344 and HIV-1Tg rat brain, the level of the pro-inflammatory cytokine, IL-1β, was elevated in the LL+L (> 9 fold) and SS+L (> 4 fold) groups. The level of the anti-inflammatory cytokine, IL-10, was elevated in the brain of both the SS+L (> 3 fold) and LL+L (> 2 fold) groups in the F344 rats, but only in the brain of the SS+L (> 2 fold) group in the HIV-1Tg rat brain (Figure 1). In the spleen of the F344 and HIV-1Tg rats, the levels of the pro-inflammatory cytokines, IL-1α, IL-1β, were elevated in both the SS+L (> 10 fold) and LL+L (> 5 fold) groups. The levels of the pro-inflammatory cytokines, IFN-γ and TNF-α, were increased in the SS+L group (> 6 fold), and the anti-inflammatory cytokine, IL-10, was higher in the SS+L (> 5 fold) and LL+L (> 7 fold) groups (Figure 1).

#### Expression profiles of chemokine and chemokine receptors in the brain and spleen of the HIV-1Tg rat following LPS treatment

**Figure 2:**
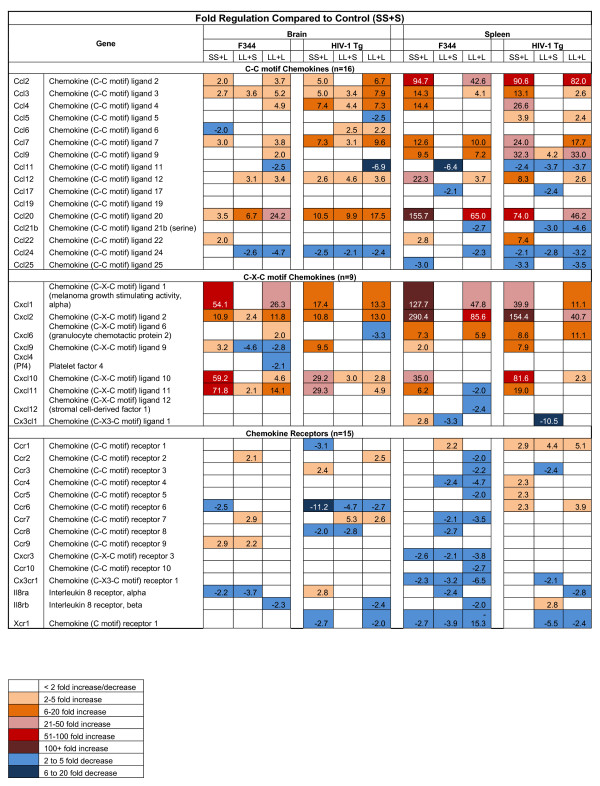
**Chemokine Expression in the Brain and Spleen of F344 and HIV-1Tg Rats**.

The gene expression profiles of various chemokines and chemokine receptors were examined in the three LPS treatment groups and compared to the control group (SS+S) in both the HIV-1Tg and F344 rats (Figure 2). The gene expression of the inflammatory cc chemokines, Ccl2, Ccl3, Ccl7, and Ccl20, and inflammatory Cxc chemokines, Cxcl1, Cxcl2, and Cxcl10, were up-regulated in the brain and spleen in the SS+L and LL+L groups of both the F344 and HIV-1Tg rats. The gene expression of the inflammatory cc chemokines, Ccl11 and Ccl24, were down-regulated in the brain of the LL+L group of both the F344 and HIV-1Tg rats, and in the SS+L and LL+L groups in the spleen of the HIV-1Tg rats.

Ten of the 15 (10/15) chemokine receptors studied were down-regulated in the LL+L group of the spleen of the F344 rats. One of 15 (1/15) and three of 15 (3/15) chemokine receptors were down-regulated in the LL+L group of the brain of the F344 and HIV-1Tg rats, respectively; ten of 15 (10/15) and two of 15 (2/15) chemokine receptors were diminished in the LL+L group of the spleen of the F344 and HIV-1Tg rats (Figure 2).

## Discussion

Endotoxin tolerance (ET) is a phenomenon in which previous exposure of cells or organisms to microbial products, such as the endotoxin, LPS, induces a transient period of hypo-responsiveness to a subsequent endotoxin challenge. Exposure to an endotoxin initiates the production of pro-inflammatory cytokines and a subsequent production of anti-inflammatory cytokines by immune cells [7,15,53,54]. ET is characterized by diminished release of pro-inflammatory cytokines, such as IL-1β, IL-1α, and TNF-α, and increased expression of anti-inflammatory cytokines, such as 1L-10 [14]. This negative feedback mechanism is important for protecting the host from tissue damage and death caused by excessive inflammation The differential expression of cytokines/chemokines in different tissues and at different times has been examined in Sprague Dawley rats to investigate the modulation of immune responses [46].

The occurrence of ET has been shown to increase the incidence of several diseases, including sepsis, pancreatitis, trauma and surgery [55]. The CDC has identified the increase of HIV-1 positive population as a major factor in the increased incidence of sepsis [44]. Studies have shown that the presence of HIV viral proteins causes a compromised immune response in HIV-1 patients [56-60]. The phenomenon of ET during HIV-1 infection has not been studied through animal models. Reid and colleagues [32] established a non-infectious HIV-1 transgenic (HIV-1Tg) rat model that expresses an HIV-1 provirus regulated by the viral promoter, but with a functional deletion of *gag *and *pol*. The characteristics of the HIV-1Tg rat include immunologic dysfunction, nephropathy, muscle wasting, skin lesions, and cataracts. We studied the systemic effect of the concurrent presence HIV-1 viral proteins and ET on the inflammatory response to bacterial endotoxin using the HIV-1Tg rat model. We examined the LPS-induced gene expression of 84 cytokines, chemokines, and their receptors in the blood, brain, and spleen of the endotoxin tolerant HIV-1Tg rat. Based on previous studies, we used two injections of a low dose of LPS (250 μg/kg) administered 9 h apart to induce ET, and a challenge injection with a high dose of LPS (5 mg/kg) 12 h later. A single exposure to a high dose of endotoxin should cause a significant increase in the levels of pro-inflammatory and anti-inflammatory cytokines compared to control [46]. In the ET state, when animals are exposed to repeated low doses of endotoxin, upon a subsequent challenge with a high dose of the endotoxin, one would expect that the increase in the pro-inflammatory cytokine levels would be lower compared to that in the single high dose exposure group [14]. To confirm that the animals were in the ET state, we measured the protein levels of inflammatory cytokines after the LPS challenge dose. Although the basal levels in the brain, spleen, and serum of the control groups (SS+S) of F344 and HIV-1Tg rats were similar, the changes in the cytokine and chemokine profiles in response to LPS were different in the HIV-1Tg and F344 rats. We found that, during ET, there was a significantly diminished expression of pro-inflammatory cytokines, such as IL-1β, IFN-γ, and TNF-α, in response to LPS (LL+L) compared to a single exposure of LPS (SS+L).

Of particular interest was the finding that the basal level of IFN-γ in the spleen was lower in the SS+S group of HIV-1Tg rats than of the F344 rats, but was significantly increased in the SS+L and LL+L groups of the HIV-1Tg rats compared to the F344 rats (Table 3). A recent study showed that IFN-γ countered ET by facilitating Toll-like receptor (TLR)-induced chromatin remodeling [61]. ET was prevented in IFN-γ pre-treated primary human monocytes, and production of pro-inflammatory cytokines, such as TNF-α and IL-6, was restored by facilitating TLR-induced chromatin remodeling [61]. It would be interesting to examine what role IFN-γ plays in the restoration of the production of pro-inflammatory cytokines at the transcriptional level in the HIV-1Tg rat compared to control.

We found that, in both the ET state and after a single exposure to LPS, there was an altered response to LPS in terms of pro-inflammatory cytokine production in the spleen of the HIV-1Tg rats compared to the F344 rats. The expression level of the pro-inflammatory cytokines, IL-1α, IL-1β, and IFN-γ, was 4- to 82-fold greater in the spleen of the HIV-1Tg rats compared to the F344 rats in both the single LPS exposure (SS+L) and ET (LL+L) groups, indicating that the presence of viral proteins may have an effect on innate immune responses. There was also a difference in the expression of cytokine receptors in the brain of the HIV-1Tg rats compared to the F344 rats in response to LPS in both the ET state and after a single exposure to LPS, suggesting that HIV-1 viral proteins may interact with or work through cytokine receptors in the brain. These data warrant further investigation into the neuroimmune effects of HIV-1 viral proteins.

During ET, the production of pro-inflammatory cytokines, such as IL-1β, IL-4, and IL-5, in response to LPS (LL+L) was diminished in contrast to that elicited by a single exposure to the endotoxin (SS+L). This altered response was seen in the spleen and serum in both the HIV-1Tg and F344 ET rats.

The spleen is an immune system organ, and, as such, one would expect that a greater number of cytokine and chemokine genes would be changed in the spleen in response to an immune challenge compared to the brain, and that is what we found in this study. In both the HIV-1Tg and F344 rats, there was a greater response to LPS in the spleen than in the brain.

Chemokines and their receptors have been implicated in the neuropathogenesis of HIV-1 infection [62]. There were significant differences in chemokine expression in response to LPS in the HIV-1Tg rats compared to the F344 rats. Enhanced levels of Ccl2 in HIV-1 patients have been associated with HIV-1-associated dementia [43]. Our results indicate that Ccl2 levels were increased to a greater extent (3- to 5-fold) in the brain of the SS+L and LL+L groups of HIV-1Tg rats versus the F344 rats, corroborating the role of upregulation of Ccl2 and its implications on HIV encephalopathy. The levels of the inflammatory CXC chemokines, Cxcl1, Cxcl2, Cxcl10, and Cxcl11, were elevated to a greater extent (0.1- to 40-fold) in the brain of the SS+L and LL+L groups of the F344 rats compared to the HIV-1Tg rats. Ccl2, Ccl7, and Ccl9, levels were increased to a greater extent in the SS+L and LL+L groups of the spleen of the HIV-1Tg rats in comparison to the F344 rats. Cxcl1 and Cxcl2 levels were elevated to a lesser extent in the spleen of the SS+L and LL+L groups of the F344 rats compared to the HIV-1Tg rats.

Chemokines and chemokine receptors define a network throughout the body, playing critical roles in immune and inflammatory responses as well as in many pathological processes, in diseases such as multiple sclerosis, Alzheimer's disease, and HIV/AIDS [63]. Cxcr4 and Ccr5 are reported to be co-receptors that mediate HIV-1 entry [64]. In our study, the gene expression of the chemokine receptors, Ccr2, Ccr3, Ccr4, Ccr5, Ccr7, Cxcr3, Ccr10, Ccr3, Cx3cr1, IL-8rβ, and Xcr1, in the spleen of the ET group (LL+L) of F344 rats were down-regulated, whereas those in the HIV-1Tg spleen were not significantly different compared to the control group (SS+S). All these receptors have been shown to function as co-receptors for HIV-1infection *in vitro *[65], which suggests that HIV-1 viral proteins may interact with these chemokine receptors *in vivo*. There is also evidence that chemokines and chemokine receptors play an important part in the signaling of neuroprotective effects in the brain [63].

In this study, we noted a distinct pattern of cytokine/chemokine expression in the brain, spleen, and serum of the HIV-1Tg and F344 rats in response to LPS, both with and without ET. Identifying these distinct cytokine/chemokine profiles may potentially be useful as indicators of the onset and/or progression of certain disease processes, such as sepsis. Further studies will be done to determine the relationship of viral protein expression to the production of cytokines and chemokines during ET.

## Conclusions

The data from our study provide a comprehensive picture of the neuroimmune responses to infection during ET, and strongly suggest that the presence of HIV-1 viral proteins may exacerbate those responses. These findings also suggest the potential use of experimentally defined cytokine/chemokine expression profiles as indicators of altered immune function in various disease states.

## Abbreviations

Abcf1: ATP-binding cassette, sub-family F (GCN20), member 1; AIDS: Acquired Immune Deficiency Syndrome; Bcl6: B-cell CLL/lymphoma 6; C3: Complement component 3; Casp1: Caspase 1; Ccl11: Chemokine (C-C motif) ligand 11; Ccl12: Chemokine (C-C motif) ligand 12; Ccl17: Chemokine (C-C motif) ligand 17; Ccl19: Chemokine (C-C motif) ligand 19; Ccl2: Chemokine (C-C motif) ligand 2; Ccl20: Chemokine (C-C motif) ligand 20; Ccl21b: Chemokine (C-C motif) ligand 21b; Ccl22: Chemokine (C-C motif) ligand 22; Ccl24: Chemokine (C-C motif) ligand 24; Ccl25: Chemokine (C-C motif) ligand 25; Ccl3: Chemokine (C-C motif) ligand 3; Ccl4: Chemokine (C-C motif) ligand 4; Ccl5: Chemokine (C-C motif) ligand 5; Ccl6: Chemokine (C-C motif) ligand 6; Ccl7: Chemokine (C-C motif) ligand 7; Ccl9: Chemokine (C-C motif) ligand 9; Ccr1: Chemokine (C-C motif) receptor 1; Ccr1: Chemokine (C-C motif) receptor 1; Ccr1: Chemokine (C-C motif) receptor 1; Ccr10: Chemokine (C-C motif) receptor 10; Ccr2: Chemokine (C-C motif) receptor 2; Ccr3: Chemokine (C-C motif) receptor 3; Ccr4: Chemokine (C-C motif) receptor 4; Ccr5: Chemokine (C-C motif) receptor 5; Ccr6: Chemokine (C-C motif) receptor 6; Ccr7: Chemokine (C-C motif) receptor 7; Ccr8: Chemokine (C-C motif) receptor 8; Ccr9: Chemokine (C-C motif) receptor 9; Cd40lg: CD40 ligand; CNS: Central nervous system; Crp: C-reactive protein, pentraxin-related; CT: Threshold cycle value; Cxcl1: Chemokine (C-X-C motif) ligand 1 (melanoma growth stimulating activity, alpha); Cx3cl1: Chemokine (C-X3-C motif) ligand 1; Cx3cr1: Chemokine (C-X3-C motif) receptor 1; Cxcl10: Chemokine (C-X-C motif) ligand 10; Cxcl11: Chemokine (C-X-C motif) ligand 11; Cxcl12: Chemokine (C-X-C motif) ligand 12; Cxcl2: Chemokine (C-X-C motif) ligand 2; Cxcl4 (Pf4): Platelet factor 4; Cxcl6: Chemokine (C-X-C motif) ligand 6 (granulocyte chemotactic protein 2); Cxcl9: Chemokine (C-X-C motif) ligand 9; Cxcr3: Chemokine (C-X-C motif) receptor 3; ET: Endotoxin Tolerance; F344: Fisher/NHsd 344 control rats; HAART: Highly active anti-retroviral therapy; HIV-1: Human immunodeficiency virus-1; HIV-1Tg: HIV-1 Transgenic; IACUC: Institutional Animal Care and Use Committee; IFN-β: Interferon beta; IFN-γ: Interferon Gamma; IL-10: Interleukin 10; IL-10rα: Interleukin 10 receptor, alpha; IL-11: Interleukin 11; IL-12: Interleukin 12; IL-13: Interleukin 13; IL-13rα1: Interleukin 13 receptor alpha 1; IL-15: Interleukin 15; IL-16: Interleukin 16; IL-17B: Interleukin 17B; IL-18: Interleukin 18; IL-1F5: Interleukin 1 family, member 5 (delta); IL-1F6: Interleukin 1 family, member 6; IL-1r1: Interleukin 1 receptor, type I; IL-1r2: Interleukin 1 receptor, type II; IL-1α: Interleukin 1, alpha; IL-1β: Interleukin 1, beta; IL-2rβ: Interleukin 2 receptor, beta; IL-2rγ: Interleukin 2 receptor, gamma; IL-3: Interleukin 3; IL-4: Interleukin 4; IL-5: Interleukin 5; IL-5rα: Interleukin 5 receptor, alpha; IL-6: Interleukin 6; IL-6rα: Interleukin 6 receptor, alpha; IL-6st: Interleukin 6 signal transducer; IL-8rα: Interleukin 8 receptor, alpha; IL-8rβ: Interleukin 8 receptor, beta; Itgam: Integrin alpha M; Itgb2: Integrin beta 2; LEA: LPS-induced leukocyte-endothelial adhesion; LPS: Lipopolysaccharide; Lta: Lymphotoxin alpha (TNF superfamily, member 1); Ltb: Lymphotoxin beta (TNF superfamily, member 3); Mif: Macrophage migration inhibitory factor; PCR: Polymerase chain reaction; RGD1561905_predicted: Complement component 5; Scye1: Small inducible cytokine subfamily E, member 1; Spp1: Secreted phosphoprotein 1; Tgfb1: Transforming growth factor, beta 1; TNF: Tumor necrosis factor (TNF superfamily, member 2); TNFrsf1a: Tumor necrosis factor receptor superfamily, member 1a; TNFrsf1b: Tumor necrosis factor receptor superfamily, member 1b; Tollip: Toll interacting protein; Xcr1: Chemokine (C motif) receptor 1

## Competing interests

The authors declare that they have no competing interests.

## Authors' contributions

NFH participated in the experimental design, coordination, tissue collection, and pilot PCR array studies, and was the primary drafter of the manuscript. XM participated in tissue collection, carried out the PCR array studies, and provided input on the manuscript. EFL carried out the cytokine/chemokine protein measurement studies, and provided input on the manuscript. SLC conceived the idea of the study, designed and coordinated the experiments and assays, and conducted blind studies, data analysis, and manuscript preparation. All authors read and approved the final manuscript.
